# Lyophilised Platelet-Rich Fibrin: Physical and Biological Characterisation

**DOI:** 10.3390/molecules26237131

**Published:** 2021-11-25

**Authors:** Nurul Aida Ngah, George J. Dias, Darryl C. Tong, Siti Noor Fazliah Mohd Noor, Jithendra Ratnayake, Paul R. Cooper, Haizal Mohd Hussaini

**Affiliations:** 1 Faculty of Dentistry, Sir John Walsh Research Institute, University of Otago, P.O. Box 56, Dunedin 9054, New Zealand; darryl.tong@otago.ac.nz (D.C.T.); jithendra.ratnayake@otago.ac.nz (J.R.); p.cooper@otago.ac.nz (P.R.C.); haizal.mh@otago.ac.nz (H.M.H.); 2 Faculty of Dentistry, Universiti Teknologi MARA, Sungai Buloh Campus, Jalan Hospital, Sungai Buloh 47000, Malaysia; 3 Department of Anatomy, School of Biomedical Sciences, University of Otago, P.O. Box 56, Dunedin 9054, New Zealand; george.dias@otago.ac.nz; 4 Craniofacial and Biomaterial Sciences Cluster, Advanced Medical and Dental Institute, Universiti Sains Malaysia, Kepala Batas 13200, Malaysia; fazliah@usm.my; 5 Faculty of Dental Medicine, Kampus A Universitas Airlangga, Surabaya 60132, Indonesia

**Keywords:** lyophilisation, platelet concentrate, platelet-rich fibrin, craniofacial regeneration, tissue engineering

## Abstract

Background: Platelet-rich fibrin (PRF) has gained popularity in craniofacial surgery, as it provides an excellent reservoir of autologous growth factors (GFs) that are essential for bone regeneration. However, the low elastic modulus, short-term clinical application, poor storage potential and limitations in emergency therapy use restrict its more widespread clinical application. This study fabricates lyophilised PRF (Ly-PRF), evaluates its physical and biological properties, and explores its application for craniofacial tissue engineering purposes. Material and methods: A lyophilisation method was applied, and the outcome was evaluated and compared with traditionally prepared PRF. We investigated how lyophilisation affected PRF’s physical characteristics and biological properties by determining: (1) the physical and morphological architecture of Ly-PRF using SEM, and (2) the kinetic release of PDGF-AB using ELISA. Results: Ly-PRF exhibited a dense and homogeneous interconnected 3D fibrin network. Moreover, clusters of morphologically consistent cells of platelets and leukocytes were apparent within Ly-PRF, along with evidence of PDGF-AB release in accordance with previously reports. Conclusions: The protocol established in this study for Ly-PRF preparation demonstrated versatility, and provides a biomaterial with growth factor release for potential use as a craniofacial bioscaffold.

## 1. Introduction

Platelet-rich fibrin (PRF) has gained popularity in craniofacial surgery and dentistry due to the absence of anticoagulant and xeno-origin components. It provides a rich reservoir of autologous growth factors (GFs) that are essential for bone regeneration, including (1) platelet-derived growth factors (PDGF), (2) transforming growth factor beta (TGFβ), and (3) vascular endothelial growth factors (VEGF), with prolonged release of these GFs to surgical areas [1,2,3]. PRF was previously developed as fresh platelet concentrates for same-day application as an adjunct for regenerative bone therapies [4]. Its low elastic modulus, limited storage capabilities, and restrictions in emergency therapy use have constrained its more widespread clinical application [5,6,7,8].

A suitable method to preserve the unique properties of PRF (i.e., growth factor release) and its microarchitecture is required to optimise its clinical application [9]. The search for new approaches to preserve the biological function of platelets is considerably important; thus, lyophilisation or a freeze-drying method was proposed to address the current drawbacks of the short clinical half-life of fresh platelet concentrates [10]. In this context, PRF lyophilisation, i.e., freezing followed by water sublimation and the subsequent removal of water vapour, was proposed as a consistent method for fabrication for an off-the-shelf product with improved stability, ready for future application [10]. Lyophilised or freeze-dried PRF (Ly-PRF) can be prepared on an autologous basis, and can be applied for treatments requiring multiple applications, such as alveolar cleft bone grafting. The lyophilisation of PRF was previously found to provide better storage stability, with a longer half-life and preservation of GFs [11]. Indeed, recent studies showed that there is justification for the development of large-scale production of Ly-PRF for future usage, particularly for bone augmentation [12,13]. There is a continuous release of platelet-derived growth factor AB (PDGF-AB) from fresh PRF. However, little is known about the preservation and release pattern of PDGF-AB in Ly-PRF, and its three-dimensional (3D) microarchitecture. 

Consequently, this paper generates lyophilised PRF (Ly-PRF), characterises its physical and biological properties, and explores the potential application of the Ly-PRF in craniofacial tissue engineering by determining: (1) the physical and morphological architecture of Ly-PRF’s using SEM, (2) the surface area and cross-sectional structure of Ly-PRF using SEM, and (3) the release kinetics of PDGF-AB using ELISA.

## 2. Results

### 2.1. Demographic Data of Donors

Five systemically healthy participants (2 males and 3 females) were enrolled. Their mean age was 22.6 ± 1.14 years. There were no health-related complications reported prior to, during, or after blood collection.

### 2.2. Morphological Analysis of Ly-PRF

**Figure 1:**
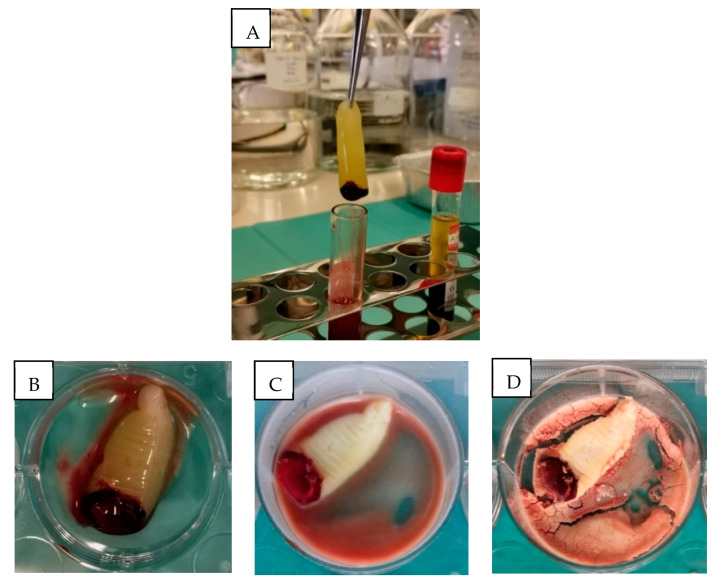
Platelet-rich fibrin (PRF) preparation. (**A**) Venous blood centrifuged for separation. PRF clot formed in the tube centre between red blood cells and platelet-poor plasma fractions. Appearance of (**B**) fresh PRF, (**C**) frozen PRF, and (**D**) lyophilised PRF.

**Figure 2:**
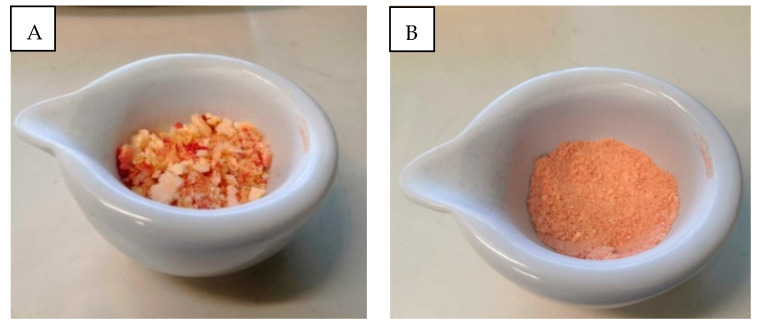
Physical appearance of lyophilised PRF. Ly-PRF (**A**) before and (**B**) after being ground into granules using a mortar and pestle.

The mean weight of PRF and Ly-PRF from the five donor volunteers is shown in Table 1. After the freeze-drying process, the Ly-PRF material resembled a sponge, with a combination of yellowish and red colouration this was consistent in structure with previous protocols [3,14]. The middle layer was carefully removed using forceps along with a thin portion of the RBC layer, and this represents the traditional PRF fraction. This material had gel-like consistency (Figure 1A,B). The isolated PRF had a minimal weight of 970 ± 0.56 mg and a maximal weight of 1675 ± 0.24 mg. The lowest weight for Ly-PRF was 104 ± 0.03 mg, and the highest weight was 367 ± 0.12 mg. Figure 1C,D display the different morphology and physical characteristics of PRF and Ly-PRF. Weight loss ranged from 70% to 91% for all PRF samples following the freeze-drying process. The difference in PRF weight before and after the freeze-drying process was statistically significant (*p* < 0.05) (Table 2). Figure 2 displays the physical appearance of Ly-PRF before and after being ground into granules using a mortar and pestle. 

### 2.3. Structural and Surface Characterisation of Ly-PRF Using SEM 

#### 2.3.1. Surface Morphological Features of Ly-PRF

**Figure 3:**
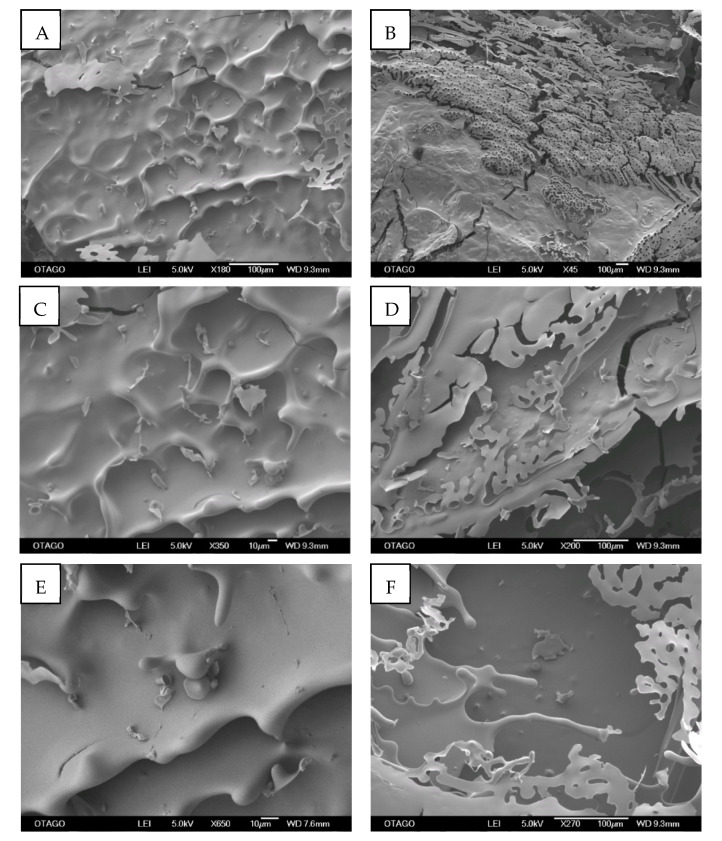
Representative scanning electron microscope (SEM) micrographs of surface of intact Ly-PRF, which showed irregular-surface topographical appearance. SEM magnifications: ((**A**) ×45, (**B**) ×180, (**C**) ×200, (**D**) ×270, (**E**) ×350, and (**F**) ×650). (Bar = 100 µm).

In general, under SEM, the surface morphology of Ly-PRF was irregular (Figure 3).

#### 2.3.2. Porous Structure of Ly-PRF

**Figure 4:**
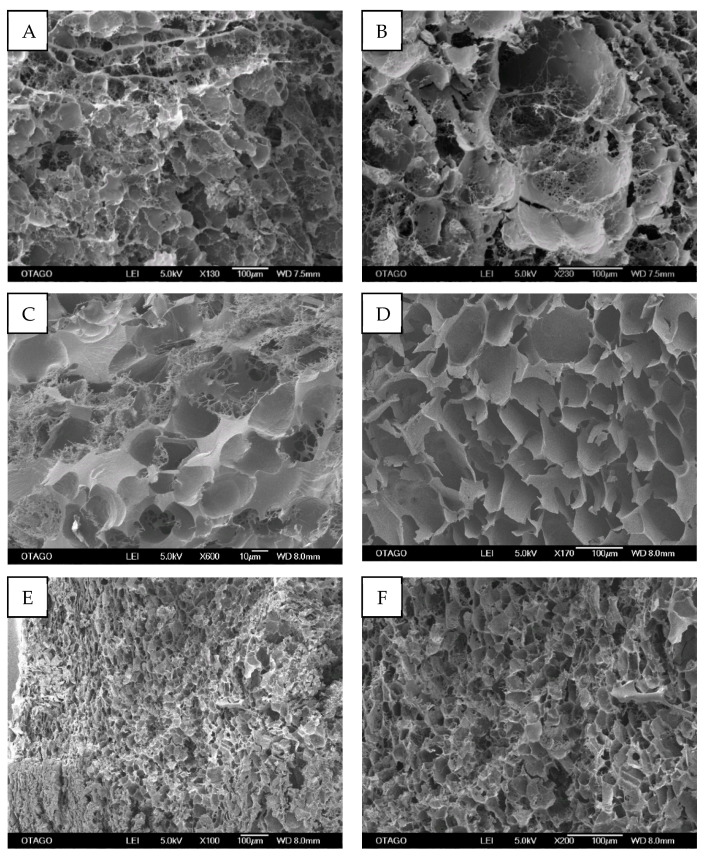
Cross-sections of intact Ly-PRF demonstrated a highly porous structure with a mixture of homogenous and heterogenous pore sizes. (SEM; original magnification: (**A**) ×130, (**B**) ×230, (**C**) ×600, (**D**) ×170, (**E**) ×100 and (**F**) ×200). (Bar = 100 µm) Images (**A**,**B**) from Donor 1, images (**C**,**D**) from Donor 2 and images (**E**,**F**) from Donor 3.

The cross-sectional images of Ly-PRF exhibited porous microarchitecture with a heterogeneous network (Figure 4). The mean pore size observed for Ly-PRF was 30.91 ± 24.44, 41.16 ± 33.80 and 17.34 ± 23.05 for the three donors, respectively (Table 2). The pore size ranged from 2.45 ± 23.05 µm to 181.24 ± 23.05 µm. Donor 3 exhibited the statistically significantly lowest average pore size among all donors (*p* < 0.05). 

#### 2.3.3. Fibrin Microarchitecture of Ly-PRF

**Figure 5:**
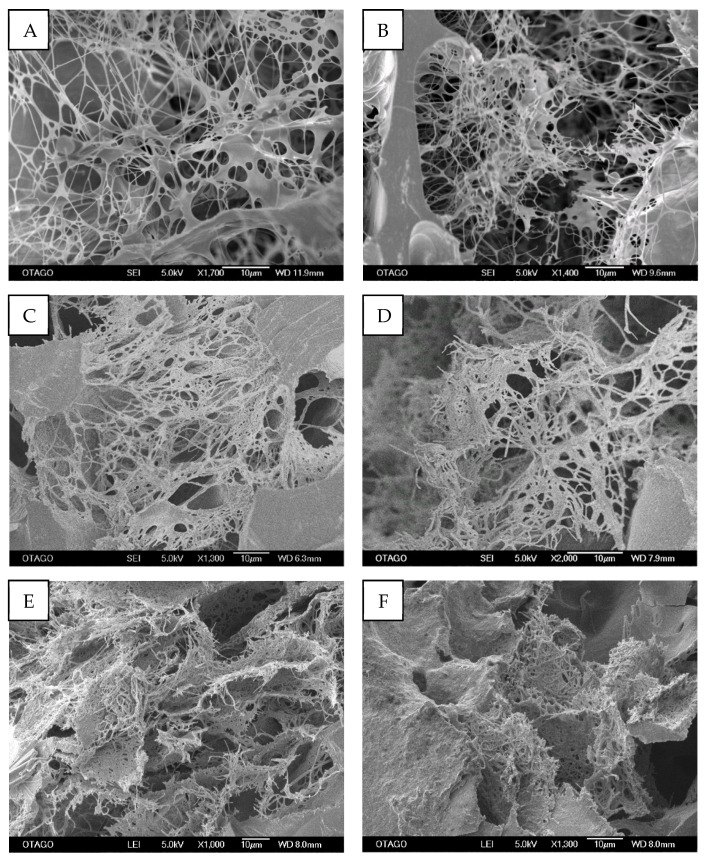
Highly dense and interconnected 3D fibrin microarchitecture in cross-sections of intact Ly-PRF. (SEM; original magnification: (**A**) ×1700, (**B**) ×1400, (**C**) ×1300, (**D**) ×2000, (**E**) ×1,000 and (**F**) ×1300). (Bar = 10 µm). (**A**,**B**) Donor 1; (**C**,**D**) Donor 2; (**E**,**F**) Donor 3.

SEM images revealed a dense 3D fibrin framework within the Ly-PRF cross-sectional view (Figure 5). The mean diameter of the fibrin fibres was 339.70 ± 133.20, 384.10 ± 151.50, 332.70 ± 133.20 nm for donor 1, donor 2 and donor 3, respectively (Table 3). Diameters ranged from 107 ± 151 nm to 649 ± 133.20 nm. Donor 2 exhibited the statistically significant largest fibrin diameter of all donors (*p* < 0.05).

#### 2.3.4. Cell Entrapment within Ly-PRF

**Figure 6:**
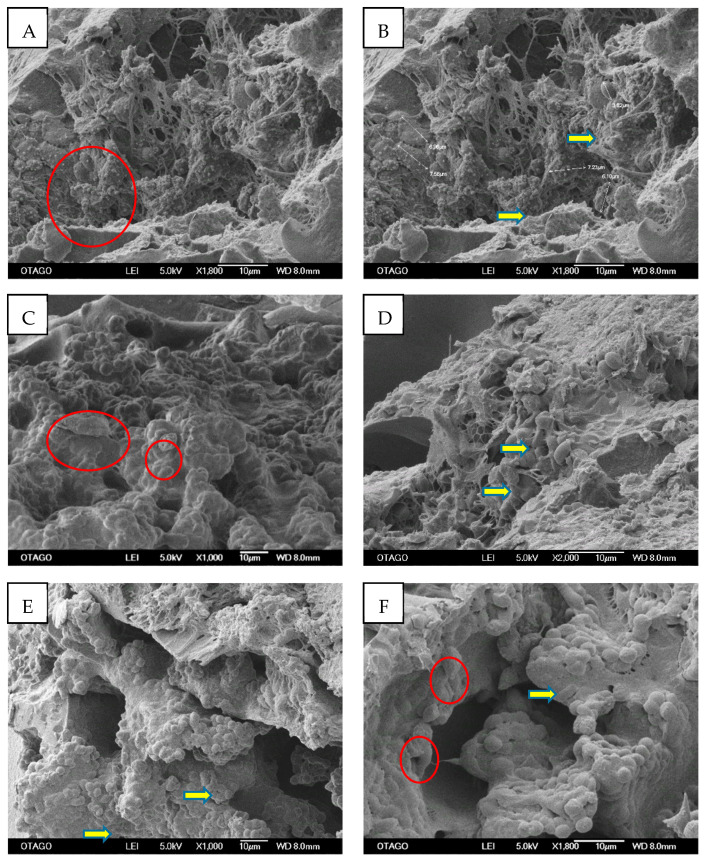
Representative SEM micrographs of cross-sections of intact Ly-PRF revealed entrapped clusters of platelets (yellow arrows) and leukocytes (red circles) within the Ly-PRF clot. (**A**) Representative image of a zone of enriched platelets with various degrees of activation. (SEM; original magnification: (**A**,**B**) ×1800, (**C**) ×1000, (**D**) ×2000, (**E**) ×1000 and (**F**) ×1800). (Bar = 10 µm).

Numerous cells were observed to be embedded in the complex three-dimensional network of the fibrin fibres. The majority of cells appeared as platelet and leukocyte clusters embedded within the fibrous structure, and were located at the border between the red and yellow clots of the Ly-PRF. Figure 6 demonstrates the clusters of platelets and leukocytes in the Ly-PRF.

### 2.4. PDGF-AB ELISA 

**Figure 7:**
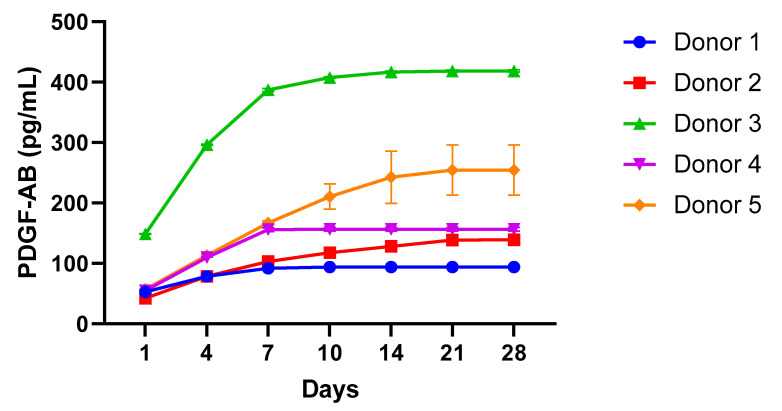
Cumulative kinetics for PDGF-AB growth factor release from five donors samples. Data presented as mean ± SD of five biological replicates (n = 5) with 2 technical replicates (n = 2).

The kinetic release of PDGF-AB growth factor in Ly-PRF was evaluated over 28 days using a commercially available enzyme-linked immunosorbent assay kit (Quantikinine ELISA (Human PDGF-AB), R&D SYSTEMS^®^, Minneapolis, MN, USA). This analysis was performed to assess the kinetic release pattern of PDGF-AB from the Ly-PRF. This timeframe was selected on the basis of previously reported studies that had performed similar analyses. The highest-level PDGF-AB growth factor release occurred on Day 1 (71.09 ± 41.24 pg/mL), and the lowest release level was on Day 28 (0.17 ± 0.32 pg/mL). A decreasing trend of PDGF-AB release was observed. Ly-PRF levels of PDGF-AB at Days 7, 10, 14, 21, and 28 were statistically lower than that on Day 1 (*p* < 0.05). On Day 1, growth factor levels were 2-, 4-, 7-, 15-, and 426-fold statistically greater than those on Days 7, 10, 14, 21, and 28, respectively. No significant differences were observed in release after Day 10 (*p* > 0.05). The cumulative average release of PDGF-AB over 4 weeks was 94.08, 139.15, 418.66, 156.55, 254.43 pg/mL, respectively (Figure 7). The PDGF-AB concentration was normalised using the relevant individual weight of Ly-PRF granules from each donor.

## 3. Discussion

The present study demonstrates the application of lyophilisation as a novel method for PRF preservation, primarily for maintaining the physical microarchitecture of PRF and preserving autologous PDGF-AB growth factor release. The Ly-PRF generated in this study displayed fundamental properties indicating its potential use as a bioactive scaffold and stimulant for bone regeneration. 

### 3.1. Ly-PRF Fabrication

The Ly-PRF fabrication protocol developed for this study was based on the method previously described by Li et al. (2014), and Kardos et al. (2018) [8,15]. This method was primarily selected due to the successful fabrication and use of Ly-PRF as a biomaterial for cranial bone regeneration both in vitro and in vivo. This approach is the first to be reported in the literature for Ly-PRF fabrication, and similar protocols were deployed in this study for the purpose of standardisation. The physical characteristics of the Ly-PRF generated in this study exhibited spongelike appearance, similar to that previously reported for Ly-PRF in the literature [8,15]. 

### 3.2. Structural Microarchitecture of Ly-PRF

The structural analysis of Ly-PRF, including the surface morphology and topography, fibrin network, cell attachment, and pore size, has thus far received limited attention. Hence, the current study provided a comprehensive description of Ly-PRF and hence contributes to a better understanding of this unique biomaterial.

#### 3.2.1. Surface Topography

Our Ly-PRF exhibited relatively rugged and irregular surfaces. These data agree with previous studies that reported that Ly-PRF exhibited a compact structure with a rough surface texture [16]. Irregular topographies of bioactive materials are frequently reported as being advantageous for bone tissue engineering, since this property facilitates osteoblast adhesion, growth, and differentiation [17,18]. Thus, our Ly-PRF demonstrated surface roughness features and properties that likely enable bone regeneration when clinically placed.

#### 3.2.2. Fibrin Network

Our results demonstrated that Ly-PRF exhibited a dense and homogeneous interconnected 3D fibrin network under SEM. The mean fibrin diameter identified in this study was larger than that reported by Bai et al. (2017), who had identified a maximal fibrin diameter of 140 ± 39 nm. This difference may have been due to their PRF being obtained from rabbit venous blood [19]. Fibrin plays a critical role in the three-dimensional structural stability of blood clots [20]. Previous studies suggested that the 3D network of the Ly-PRF is the main factor in providing the sustained release of growth factors from this unique biomaterial [21,22]. The prominent fibrin architecture of our Ly-PRF may have enabled a continuous and gradual release of growth factors throughout the study period. Furthermore, the unique property of the interconnected fibrin network structure in PRF may also protect growth factors from proteolysis. Hence, growth factors may sustain their function for a comparatively longer time period to enable their efficient promotion of bone regeneration [23]. Moreover, the solid walls around the pore (Figure 5) may have consisted of a highly polymerised and condensed fibrin matrix that surrounded the dense fibrin fibres. This structure arises due to the mechanical centrifugal and mild compressive forces that are generated during the application of Choukroun’s PRF protocol. A similar structure was previously reported by Dohan Ehrenfest et al. (2010) [14]. 

#### 3.2.3. Cell Distribution

Clusters of morphologically consistent cells of platelets and leukocytes were identified within the Ly-PRF in concordance with previous reports [20]. The majority of identified cells were morphologically consistent with platelets and leukocyte clusters, and were located between the junction of the Ly-PRF and the red blood clot. This finding agrees with previous studies that reported a maximal platelet/leukocyte density apparent within the first millimetre of the yellow clot, immediately adjacent to the red blood clot fraction [14,24]. These studies highlighted the importance of having a thorough understanding of PRF clot design in order to obtain optimal clinical outcomes when using this material. Thus, it is crucial to maintain a minimal red blood clot layer at the PRF clot end location in order to obtain the highest number of platelets and leukocytes. Platelet distribution in PRF matrices is heavily influenced by centrifugal force, rotor type, and PRF fabrication protocols [25]. Leukocytes contribute significantly to PRF’s antibacterial properties and are a distinctive feature of the material, which is why it is also referred to as leukocyte-and platelet-rich fibrin (LPRF). This terminology emphasises the importance of leukocytes and indicates their significance to PRF success for stimulating bone regeneration [22,24,26,27]. 

#### 3.2.4. Porosity 

Ly-PRF is a highly porous material with an average pore size diameter of 29.80 ± 27 µm. The mean pore sizes identified in our study were larger than those previously reported [8,15]. Li et al. (2014) observed that the mean diameter of the Ly-PRF bioscaffold used in their research for craniofacial regeneration was 8.06 ± 0.31 µm [7]. Kardos et al. (2018) reported that their Ly-PRF possessed a more compact structure with considerably fewer pores than that of Li et al. (2014). The larger mean pore diameter detected in our investigation may have been due to the mean of multiple series of Ly-PRF sections performed along the length of the Ly-PRF. Pore diameters varied according to the sectioned area. This agreed with a previous study that indicated that the area in closest proximity to the red blood cell (RBC) layer exhibited a more compact structure compared with the plasma layer [19].

An earlier study also reported that Ly-PRF was nearly twice as effective as fresh PRF at stimulating mineralisation. This beneficial property was attributed to the increased pore size in the Ly-PRF, which was 13 times greater than that found in fresh PRF [8]. Additionally, increased pore size enhances tissue engineering features, including promoting cell adhesion, proliferation, and tissue-repair-related gene expression. Therefore, our data support the notion that the lyophilisation process preserves PRF’s microarchitecture while simultaneously enhancing its tissue engineering properties, such as increased pore diameter. 

### 3.3. Growth Factors

Transforming growth factors beta-1 (TGF-β1), platelet-derived growth factors (PDGF), and vascular endothelial growth factors (VEGF) are three major growth factors present in PRF [9,22]. This study focused primarily on PDGF-AB, as it is one of the most highly released growth factors from PRF. PDGF-AB promotes tissue healing by accelerating collagen production, hence increasing wound strength and initiating callus development [20]. Additionally, it can attract and stimulate bone progenitor cells, which is critical for bone regenerative therapies in maxillofacial and craniofacial surgery [28,29]. During the early stages of healing, PDGF-AB attracts inflammatory cells, such as macrophages and mesenchymal stem cells (MSCs), to the injury site [30]. It can also stimulate local cell proliferation, differentiation, and extracellular matrix formation [21,27,31]. In our study, we demonstrated the presence and sustained release of the PDGF-AB in Ly-PRF following a freeze-drying process. PDGF is resilient to extreme heat, pH, and several proteases due to the presence of 8 cysteine residues per chain in disulphide bonds [30]. Our data demonstrated a similar pattern of PDGF-AB release from Ly-PRF in our five donor samples over 28 days. Our results also indicated that there was a gradual release from the onset of the study, which subsequently plateaued over time. This kinetic release pattern agrees with a recent study of Ly-PRF [9,32]. However, our overall PDGF-AB quantities were lower than those previously reported [32]. These data indicate that the complexity and heterogeneity of this biomaterial significantly varies between individuals.

The main issue concerning platelet concentrate technologies is not the quantity of growth factors present but rather the cells, 3D fibrin network, and growth factor types that are integrated into the final product. Furthermore, the prolonged release of growth factors enables a more effective bone repair process. The importance of PDGF-AB in the early stages of bone regeneration was highlighted [33]. Ly-PRFs may be favourable for bone regeneration, where Ly-PRF can be used as an autologous growth factor reservoir or as a non-weight-bearing autologous bioscaffold within craniofacial tissue.

### 3.4. Ly-PRF Tissue Bank Potential and Future Recommendations

Improvements in medical technologies have resulted in greater quantities and a broader range of tissue banking being available in order to enhance patients’ quality of life [34]. The potential benefit of the tissue bank for platelet concentrate allografts was proven in vivo, and in clinical trials in plastic and reconstructive surgery applications. This technique is appropriate for the treatment of chronic wounds [35,36]. The combination of tissue-banking and tissue-engineering technologies to develop biomaterials for regenerative therapy has an ever-increasing chance of success [37]. Ly-PRF exhibited many of the necessary key characteristics for a biomaterial for tissue-engineering purposes. Therefore, future research should explore other relevant properties of this biomaterial as a potential source for tissue bank platelet concentrate. 

Indeed, Ly-PRF displayed significant versatility as a promising biomaterial for craniofacial regeneration. The presence and persistence of PDGF-AB release in Ly-PRF were demonstrated, and its high porosity and interconnected fibrin network indicate its potential as a bone scaffold. Furthermore, as it is naturally derived, it can provide an effective and personalised approach for tissue repair that can be archived for future use [38,39]. The mean pore size of 29.80 ± 27 µm and degradability of Ly-PRF make it less suited for use as a craniofacial scaffold on its own. However, in combination with collagen-chitosan membranes, poly-lactic-co-glycolic acid (PLGA) with nanohydroxyapatite and polyvinyl alcohol hydrogel scaffolds, its application may be significantly enhanced for bone regeneration [7,9,38]. Consequently, further research to determine the physicochemical and biological characteristics of composite biomaterials that incorporate Ly-PRF are necessary to optimise it for clinical application.

### 3.5. Study Limitations

A limitation of this study is that we focused primarily on PDFG-AB. Therefore, future research should emphasise additional GFs present in the LyPRF, including PDGF-AA, PDGF-BB, TGF-B1, and VEGF. Furthermore, future studies should explore the quantity and viability of the cellular content (particularly platelet and leukocyte) to determine its role in bone regeneration and antibacterial activity of the LyPRF. Nevertheless, additional research is required to ascertain if there is a need for the sterilisation of this material prior to clinical application.

## 4. Materials and Methods

### 4.1. Ethical Approval and Māori Consultation

Ethical approval was obtained from the University of Otago Human Ethics Committee (Health) (approval number H19/057) and the study was conducted in full accordance with the World Medical Declaration of Helsinki. Consultation with the Māori was undertaken with the Ngāi Tahu Research Consultation Committee. 

### 4.2. Subjects Selection

PRF was prepared from human venous blood obtained from five healthy volunteers [23]. All subjects enrolled in this research gave informed consent, and were provided with an explanation of the research protocol and the expected benefits from this study, and future potential clinical use by the community. Inclusion criteria for this study were: age range from 25 to 35 years old, being fit and healthy, nonsmokers, and with no history of recent aspirin intake or anticoagulant drugs. Participants who had recently taken antiplatelet or anticoagulant medication, had any chronic medical issues, or were smokers were excluded from the study. 

### 4.3. Fabrication of Lyophilised Platelet-Rich Fibrin 

Venous blood was taken without anticoagulant in a 10 mL glass tube (A-PRF™ by Choukroun, Auckland, New Zealand) and was immediately centrifuged according to Choukroun’s protocol. Briefly, the blood was centrifuged at 400× *g* (approximately 3000 rpm) for 10 min using an Eppendorf Centrifuge 5810 R [14,16]. Following centrifugation, the blood separated into three layers distinguished by the following: (1) a transparent and acellular layer characterised the upper layer, (2) the middle layer was the PRF (fibrin buffy coat) layer, and (3) the third layer represented red blood cells. The middle layer was removed from the centrifuge tube using sterile forceps, and separated from the third layer using sterile scissors with a thin layer of RBC being retained. Freshly prepared PRF matrices were carefully compressed using a stainless-steel PRF compression device obtained from A-PRF kit (Ostralos Ltd., Auckland, New Zealand). To prepare Ly-PRF, intact fresh PRF was frozen and stored at −80 °C for 30 min before being freeze-dried (FreeZone^®^ Triad Freeze Dry System Models 740000 Series, Labconco, Kansas City, MO, USA) overnight at −51 °C to produce Ly-PRF [8,15]. Samples were ground into granules using a mortar and pestle before storage at 4 °C [7,9,40].

### 4.4. Quantification of Growth Factors in Lyophilised PRF

PDGF-AB was selected as a representative molecule to quantify the release of growth factors from the fabricated Ly-PRF. The weight of Ly-PRF granules was based on the mean weight (mg) of each intact Ly-PRF from each donor [41]. Ly-PRF granules were incubated in 4 mL of Dulbecco’s Modified Eagle’s Medium (DMEM) in a cell culture flask at 37 °C and with 5% CO_2_ for 28 days to evaluate the kinetics of PDGF-AB growth factor release. We collected 4 mL of conditioned medium at a range of time-points (Days 1, 4, 7, 14, 21, and 28), which was replaced with an equal volume of DMEM [21,32,41]. Prior to using an enzyme-linked immunosorbent assay (ELISA) kit (Quantikine ELISA (Human PDGF-AB), R & D SYSTEMS^®^, Minneapolis, MN, USA), samples were stored at −80 °C. All reagents, working standards, sample preparation, and growth factor quantification were performed according to manufacturer instructions. Results are presented as the mean ± standard deviation and were analysed for statistically significant differences [7].

### 4.5. Scanning Electron Microscopy (SEM) Analysis

SEM analysis was conducted to characterise the fabricated Ly-PRF microarchitecture. SEM enabled the evaluation of the materials’ surface, fibrin distribution, pore sizes assessment and cell entrapment within the fibrin network. It was also used to evaluate the overall surface topography and cross-sectional architectures of Ly-PRF. After 1 h of fixation in 2.5% glutaraldehyde (G6257-Sigma-Aldrich, St. Louis, MO, USA), Ly-PRFs were dehydrated using graded ethanol [14,42]. The Ly-PRF was gold-sputter-coated before being observed by SEM (JOEL, JSM-6700F, Tokyo, Japan), as shown in Figure 3. Fibre and pore sizes were obtained by measuring at least 100 fibres and pores, respectively, from three distinctive images using ImageJ software (Bethesda, MD, USA) to obtain their mean diameter and pore sizes [31].

### 4.6. Statistical Analysis

All statistical analyses were conducted using statistical analysis software PRISM (GraphPad Prism 6, San Diego, CA, USA). Data for the experiments are expressed as mean ± standard deviation (SD). Each experiment was performed at least twice and prepared (n = 3) for each volunteer at designated intervals. Repeated-measures within-group ANOVA was performed to compare the concentration of the PDGF-AB concentration at a 7 time-point measurement within the 28 days. Comparison for PDGF-AB concentration was performed using repeated-measures between-group ANOVA (two-way) with Tukey’s post hoc correction for multiple comparisons being undertaken. Moreover, the comparison of pore sizes and fibrin diameters between the three donors were conducted using ANOVA (one-way) with Turkey’s post hoc test performed for multiple comparison. A *p* value < 0.05 was considered to be statistically significant.

## 5. Conclusions

The protocol established in this study for generating Ly-PRF enables the production of a platelet concentrate with continuous growth factor release for bone regeneration applications in a simple, cost-effective, and organic approach. The Ly-PRF developed via the lyophilisation method demonstrated versatility as a potential biomaterial for use as a craniofacial bioscaffold. Apart from its unique properties as a reservoir for growth factor PDGF-AB, Ly-PRF exhibited fundamental scaffold properties. Future research should explore the properties of this material as a prospective source for the tissue banking of platelet concentrate.

## Figures and Tables

**Table 1:** Comparative weight of PRF and Ly-PRF.

Donor Samples	PRF (mg) (Mean ± SD)	Ly-PRF (mg) (Mean ± SD)
1	970 ± 0.56	284 ± 0.32
2	1675 ± 0.24	367 ± 0.12
3	1153 ± 0.55	104 ± 0.03
4	1295 ± 0.39	281 ± 0.15
5	1234 ± 0.68	274 ± 0.24

**Table 2:** Comparative Ly-PRF pore sizes.

Donor Samples	Pore Sizes (µm) (Mean ± SD)
1	30.91 ± 24.44
2	41.16 ± 33.80
3	17.34 ± 23.05

**Table 3:** Comparative Ly-PRF fibrin diameter.

Donor Samples	Fibrin (nm) (Mean ± SD)
1	339.70 ± 133.20
2	384.10 ± 151.50
3	332.70 ± 133.20

## Data Availability

The data presented in this study are available on request from the corresponding author.
